# Interneuron function and cognitive behavior are preserved upon postnatal removal of Lhx6

**DOI:** 10.1038/s41598-022-09003-4

**Published:** 2022-03-22

**Authors:** Lars Voss, Marlene Bartos, Claudio Elgueta, Jonas-Frederic Sauer

**Affiliations:** Institute of Physiology I, Medical Faculty, University of Freiburg, Freiburg, Germany

**Keywords:** Neuronal development, Cellular neuroscience

## Abstract

LIM homeobox domain transcription factor 6 (Lhx6) is crucial for the prenatal specification and differentiation of hippocampal GABAergic interneuron precursors. Interestingly, Lhx6 remains to be expressed in parvalbumin-positive hippocampal interneurons (PVIs) long after specification and differentiation have been completed, the functional implications of which remain elusive. We addressed the role of adult-expressed Lhx6 in the hippocampus by knocking down Lhx6 in adult mice (> 8 weeks old) using viral or transgenic expression of Cre-recombinase in Lhx6^loxP/loxP^ mice. Late removal of Lhx6 did not affect the number of PVIs and had no impact on the morphological and physiological properties of PVIs. Furthermore, mice lacking Lhx6 in PVIs displayed normal cognitive behavior. Loss of Lhx6 only partially reduced the expression of Sox6 and Arx, downstream transcription factors that depend on Lhx6 during embryonic development of PVIs. Our data thus suggest that while Lhx6 is vitally important to drive interneuron transcriptional networks during early development, it becomes uncoupled from downstream effectors during postnatal life.

## Introduction

GABAergic interneurons provide local synaptic inhibition to dendritic and perisomatic compartments of principal cells. These cells thus control excitation of principal neurons, shape dendritic integration of incoming excitatory signals, and support the generation of network oscillations^1–3^. These diverse functions are realized by specialized interneuron classes which target different domains of principal neurons. Parvalbumin-expressing interneurons (PVIs) contact the perisomatic region and axon initial segment of principal cells thereby exerting powerful control of principal cell activity.

PVI precursor neurons are mostly generated in the medial ganglionic eminence (MGE), with a smaller contribution from the preoptic area^4^. During embryonic development, postmitotic PVI precursors leave the MGE, migrate tangentially to the cortex, and reach their final position in deep and superficial layers of the developing neocortex and hippocampus by radial migration^5^. After migration to the cortex, some interneuron precursors differentiate into PVIs and acquire their mature physiological and morphological properties.

Interneuron development is controlled by transcription factors which are neuron-type specific. In the MGE, Nkx2.1 induces the expression of LIM homeobox transcription factor 6 (Lhx6), which controls key features of interneuron development. Prenatal knockout of Lhx6 substantially delays tangentially migration of MGE-derived interneurons to the most dorsal parts of the telencephalon at embryonic day 14.5 and results in an abnormal distribution of MGE-derived interneurons in cortex and hippocampus to superficial and deep layers^6,7^. In addition, Lhx6 triggers a genetic program driving MGE precursors towards a PVI and somatostatin-positive interneuron (SOMIs) fate. Prenatal loss of Lhx6 results in drastically reduced numbers of PVIs and SOMIs in the neocortex and hippocampus^6^ and is accompanied by decreased inhibition in form of fewer spontaneous inhibitory postsynaptic currents in the dentate gyrus^8^ (DG). Consistently, Lhx6-deficient MGE cells transplanted into wildtype mice develop a late-spiking action potential phenotype reminiscent of caudal ganglionic eminence-derived (CGE) interneurons rather than PVI/SOMI typical firing responses^9^. Furthermore, in Lhx6-deficient mice transcription of PV and SOM is downregulated at postnatal day 15^10^. Finally, overexpression of Lhx6 in human pluripotent stem cells produces functional PVIs and SOMIs, demonstrating the importance of the Lhx6 transcription factor for the specification of MGE-migrating precursors as PVIs^11^.

Interestingly, the Lhx6 protein is still present in PVIs during adulthood^6^. Neuronal migration, cell-type specification and differentiation are completed at that age, raising the question what role Lhx6 might play in adult interneurons. Here, we analyzed the function of Lhx6 in adult interneurons using different strategies to remove Lhx6 from hippocampal and cortical GABAergic cells in mature mice. Our results indicate unaltered numbers as well as unchanged physiological properties of PVIs in the absence of Lhx6. Furthermore, mature mice with Lhx6-deficient PVIs retain normal cognitive function. Interestingly, expression of downstream target proteins that depend on Lhx6 during early development is only slightly affected in adult mice lacking Lhx6. Our data thus show that adult PVIs lacking Lhx6 can maintain their functional properties and suggest that downstream transcriptional networks become functionally uncoupled from Lhx6 at postnatal stages.

## Results

### Selective knock-down of Lhx6 in adult mice

**Figure 1:**
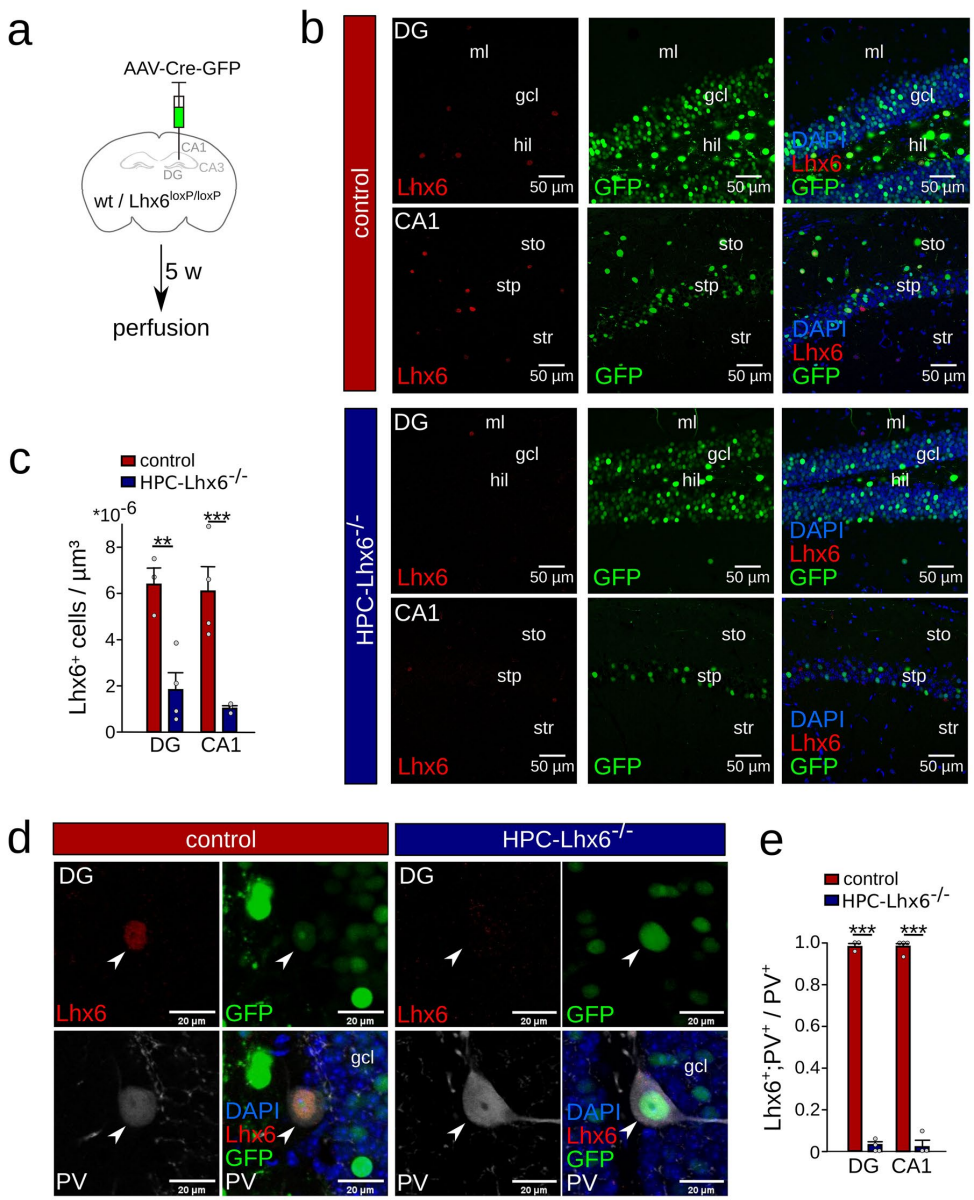
Removal of Lhx6 from adult PVIs. (**a**) Schematic of the experimental procedure used to remove Lhx6 from PVIs. Stereotaxic injections of adeno-associated viruses encoding Cre-recombinase were targeted to the hippocampus of mice carrying two alleles of the Lhx6 gene flanked by loxP-sites (HPC-Lhx6^−/−^ mice) or wildtype control mice. (**b**) Confocal image stacks of the dentate gyrus (DG) and CA1 showing the expression of Lhx6 (red), Cre-GFP (green), and DAPI (blue) in control (top) and HPC-Lhx6^−/−^ mice (bottom). Gcl: granule cell layer; hil: hilus; ml: molecular layer; stp: stratum pyramidale; sto: stratum oriens; str: stratum radiatum. (**c**) Quantification of the number of Lhx6-positive neurons revealed a significant reduction in HPC-Lhx6^−/−^ compared to control mice. (**d**) Examples of the colocalisation of Lhx6 (red), Cre-GFP (green) and parvalbumin (PV, grey) in the DG. Lhx6 immunoreactivity is absent in the majority of PVIs in HPC-Lhx6^−/−^ mice. (**e**) Quantification of the colocalisation of Lhx6 and PV in Cre-GFP-positive neurons. Data are mean ± sem. 3–4 mice were used per group. Data points show mouse averages. **p < 0.01, ***p < 0.001. Schematic in (**a**) was created with Inkscape 0.92 (www.inkscape.com).

The Lhx6 transcription factor remains to be expressed in MGE-derived interneurons after cells fully integrate into the hippocampal circuit^12,13^. To understand the functional relevance of Lhx6 in the adult hippocampal circuitry, we injected adeno-associated viruses encoding Cre recombinase fused to green fluorescent protein (AAV9-Cre-GFP) into the hippocampus of adult Lhx6^loxP/loxP^ (HPC-Lhx6^−/−^ mice) or wildtype control mice (Fig. 1a,b). Titration experiments revealed that a viral concentration of ~ 2 × 10^10^/ml was ideally suited to express Cre-GFP without affecting neuronal survival, while higher viral titers resulted in observable cell loss in the infected region (Supplementary Fig. S1). Confirming previous results^12,13^, we observed robust expression of Lhx6 in the hippocampus of adult wildtype mice (6.4 ± 0.1 × 10^–6^ and 6.1 ± 0.1 × 10^–6^ Lhx6^+^ cells/µm^3^ in DG and CA1 respectively, 13 week old mice, Fig. 1b,c, Supplementary Fig. S2). Lhx6 was expressed in the majority of PVIs (98.6 ± 1.4% and 98.3 ± 1.7% of PV^+^/Lhx6^+^ in DG and CA1, respectively, n = 1–2 slices per animal, N = 4 and 3 mice, Fig. 1d,e). Therefore, perisomatic targeting PVIs continue to express Lhx6^+^ during adulthood and comprise a significant proportion of cells expressing this transcription factor in the adult hippocampus (29.8 ± 2.7% and 49.2 ± 12.0% of Lhx6^+^ are PVIs in DG and CA1, respectively, data not shown).

Five weeks after viral injections transgenic mice showed a strong reduction in the number of Lhx6^+^ cells in both the DG and CA1 regions compared to wildtype mice that received the same viral injection (from 6.4 ± 0.7 × 10^–6^ to 1.8 ± 0.7 × 10^–6^ and from 6.1 ± 1 × 10^–6^ to 1.1 ± 0.1 × 10^–6^ cells/µm^3^ in DG and CA1, respectively, p = 0.008 and 0.001, unpaired t-tests, n = 2–3 slices per animal, N = 4 and 3 mice, Fig. 1b,c). After viral knockout of the Lhx6 gene the Lhx6 transcription factor was largely absent from PVIs (DG: 98.6 ± 2.4% vs. 2.1 ± 2.7%, p < 0.001; CA1: 98.3 ± 3.3% vs. 3.15 ± 5.5% PV and Lhx6 coexpression, p < 0.001, unpaired t-tests, n = 1–2 slices per animal, N = 4 and 3 mice, Fig. 1d,e). These data indicate the reliable knock-down of Lhx6 protein from adult hippocampal PVIs in HPC-Lhx6^−/−^ mice.

### Lhx6 is not required for the survival of adult PVIs

**Figure 2:**
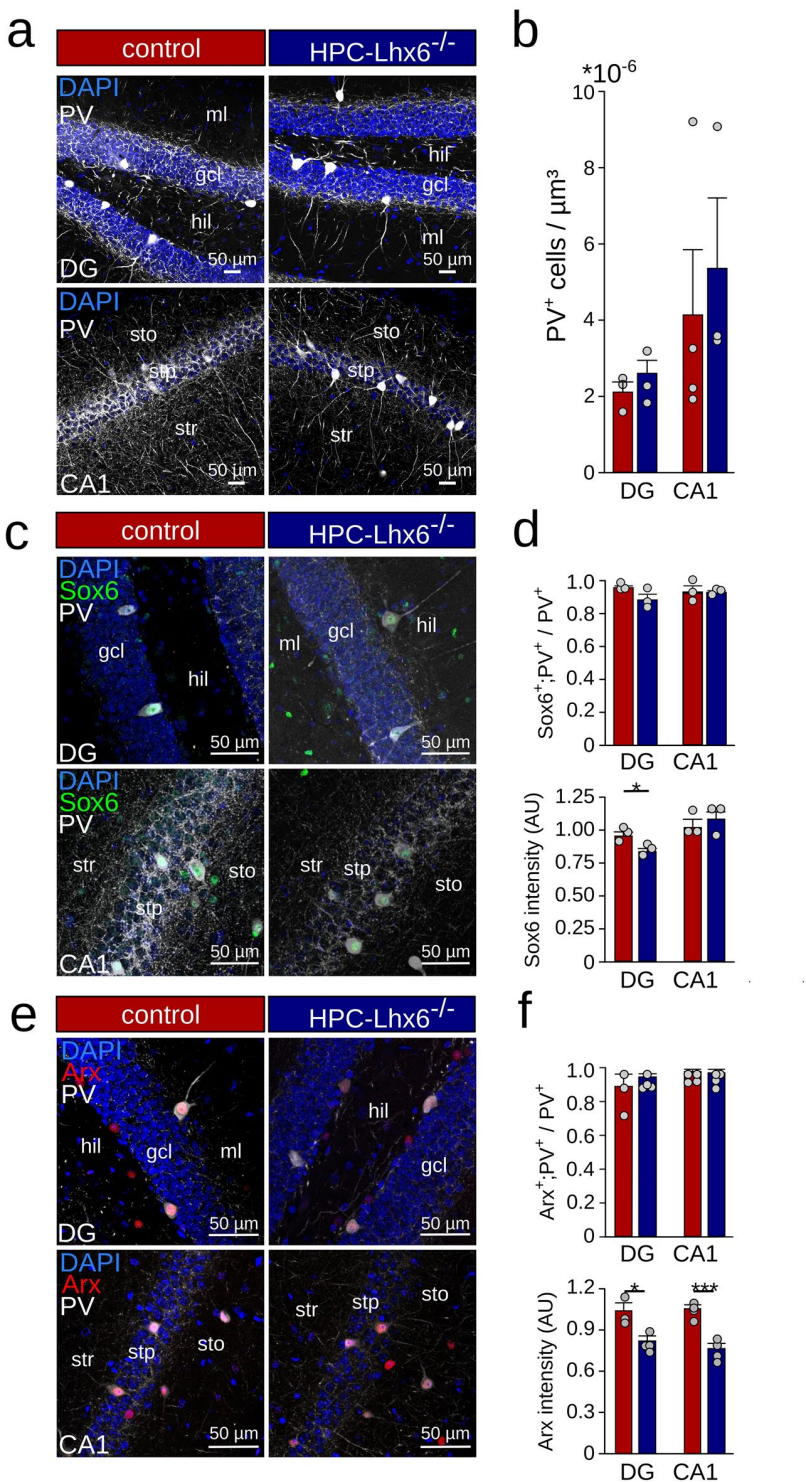
Intact downstream transcription factor expression upon removal of Lhx6 from PVIs. (**a**) Confocal image stacks of PV-staining (white) and DAPI (blue) in the dentate gyrus (DG, top) and CA1 (bottom) were used to quantify the number of PVIs in the hippocampus. *Gcl* granule cell layer, *hil* hilus, *ml* molecular layer, *sto* stratum oriens, *stp* stratum pyramidale, *str* stratum radiatum. (**b**) Quantification of the PVI density in control (red) and HPC-Lhx6^−/−^ (blue) mice revealed no significant difference between groups. (**c**) Confocal image stacks of PV (grey), Sox6 (green), and DAPI labelling (blue) in control and PV-Lhx6^−/−^ mice. (**d**) Quantification of the colabelling of Sox6 and PV revealed no difference in the number of colabelled neurons (top) but reduced expression levels in the DG of PV-Lhx6^−/−^ mice (bottom). (**e**) Same as (**c**) but with staining for the transcription factor Arx (red). (**f**) Quantification of the costaining of Arx and PV revealed no difference in the number of colabelled neurons (top) but reduced expression levels in PV-Lhx6^−/−^ mice (bottom). Data are mean ± sem. Data points show mouse averages. *p < 0.05, ***p < 0.001.

Besides being fundamental for interneuron specification and migration, Lhx6 controls the rate of apoptotic cell death of MGE-derived immature interneurons^10^. To assess whether Lhx6 is required for the survival of adult hippocampal interneurons, we quantified the density of PVIs upon viral knock-down of Lhx6. We detected no significant difference in PVI density between control and HPC-Lhx6^−/−^ animals in both DG and CA1 (DG: 2.1 ± 0.5 × 10^–6^ vs. 2.6 ± 0.7 × 10^–6^ PV^+^ cells/µm^3^, p = 0.334, unpaired t-test; CA1: 4.1 ± 3.4 × 10^–6^ vs. 5.3 ± 3.2 × 10^–6^ PV^+^ cells/µm^3^, p = 0.4, Mann–Whitney U test, n = 1–2 slices per animal, N = 4 and 3 mice, Fig. 2a,b).

### Limited impact of adult knockout of Lhx6 on downstream transcription factors

Lhx6 drives the expression of genes crucially involved in regulating interneuron development. During early development, Lhx6 controls the expression of Sox6, whose knockdown results in an immature physiological phenotype in PVIs^14^. Given the central role of Sox6 on the maturation of fast-spiking interneurons, we asked whether Lhx6 is still required to maintain Sox6 expression in adult PVIs. In DG and CA1 of adult control mice, nearly all PVIs expressed Sox6 (DG: 95.7% expression, CA1: 93.2%, n = 2–3 slices per animal, N = 3 mice each, Fig. 2c,d). This proportion remained unchanged upon removal of the Lhx6 gene during adulthood in both DG and CA1 regions (DG: 88.3%, CA1: 92.9%, p = 0.113 and p = 0.942, unpaired t-tests, n = 2–3 slices per animal, N = 3 mice each, Fig. 2c,d). Nevertheless, a small but significant decline in Sox6 fluorescence intensity was observed in DG but not in and CA1 (DG: 0.96 ± 0.03 vs. 0.84 ± 0.02, p = 0.038; CA1: 1.03 ± 0.05 vs. 1.08 ± 0.07, p = 0.491, unpaired t-tests, n = 2–3 slices per animal, N = 3 mice each, Fig. 2d).

Having identified that Sox6 remains to be expressed after loss of Lhx6 in adult PVIs, we asked whether other downstream targets of Lhx6 might become transcriptionally uncoupled from Lhx6 as well. We tested the expression of Arx, which is controlled by Lhx6 during development and controls tangential migration of interneurons to the cortex^9^. Arx^−/−^ mice show reduced PV expression and epilepsy^9^. Similar to our findings on Sox6, immunostaining revealed that nearly all PVIs expressed detectable Arx levels in control and HPC-Lhx6^−/−^ mice (~ 98% and ~ 97%, respectively, Fig. 2e,f). However, in HPC-Lhx6^−/−^ mice, Arx intensity in PVIs normalized to Arx-expression in PV-negative neurons was significantly reduced by ~ 21% in the DG (1.04 ± 0.06 vs. 0.82 ± 0.04, p = 0.015, unpaired t-test, n = 1 slice per animal, N = 3 and 4 mice) and by ~ 28% in CA1 (1.05 ± 0.03 vs. 0.76 ± 0.04, p < 0.001, unpaired t-test, n = 1 slice per animal, N = 4 mice each, Fig. 2e,f). These data suggest that while Lhx6 is not necessary for the continued expression of Sox6 and Arx, its removal leads to reduced expression of downstream transcription factors in the adult mouse.

### Unaltered physiological and morphological properties of adult Lhx6-deficient PVIs

**Figure 3:**
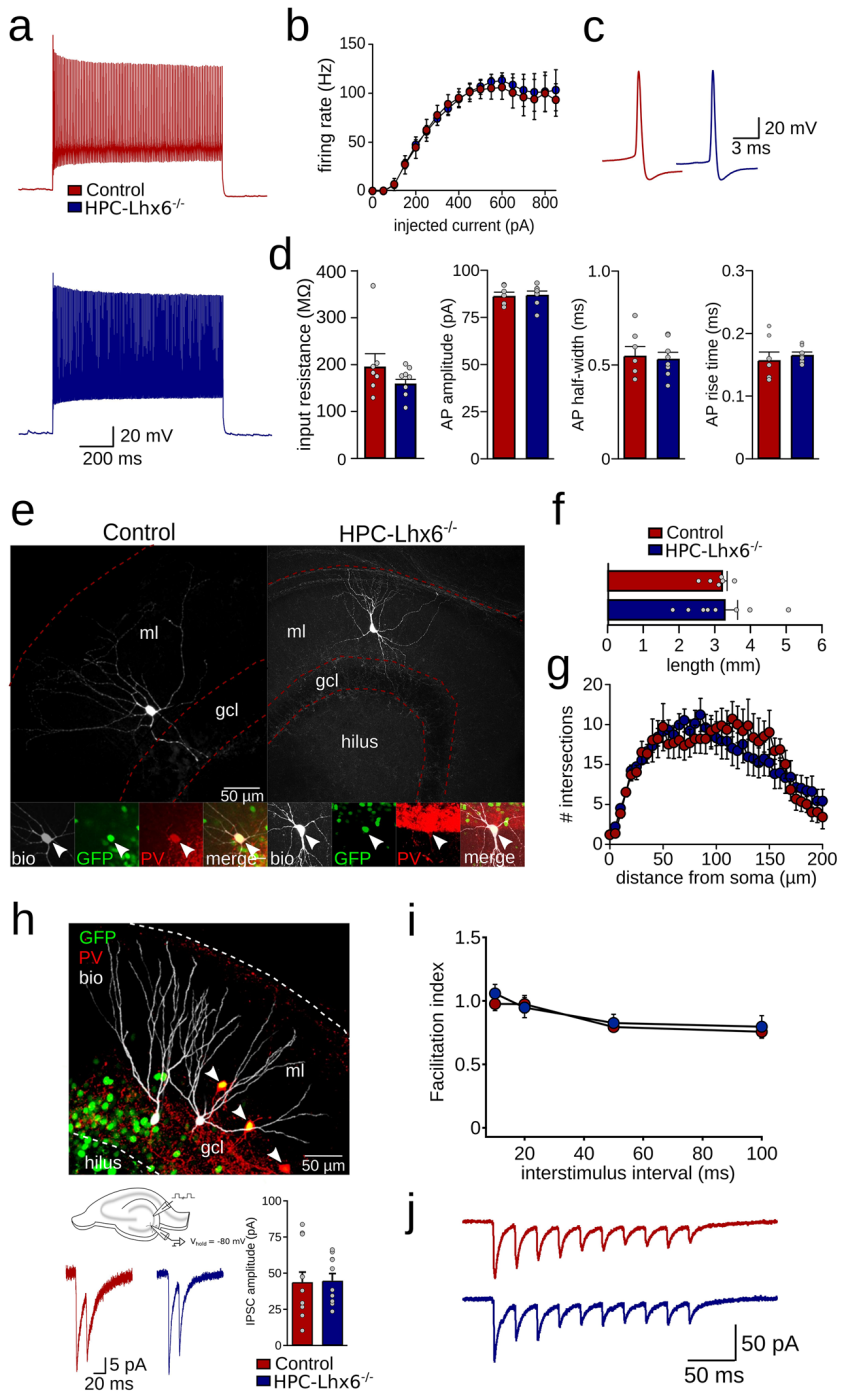
Intact physiological and morphological properties of DG fast-spiking interneurons upon adult Lhx6-knockdown. (**a**) Firing response of a control (top, red) and HPC-Lhx6^−/−^ fast-spiking interneuron (bottom, blue) in response to somatic current injection. Recordings were targeted to the DG. (**b**) Average firing rate as a function of injected current revealed that action potentials (APs) can be reliably evoked in fast-spiking interneurons of HPC-Lhx6^−/−^ mice. (**c**) Individual AP waveforms of the cells shown in (**a**). (**d**) Unchanged input resistance measured by somatic voltage application, AP amplitude, AP half-width and AP rise time. (**e**) Confocal image stacks of fast-spiking interneurons filled with biocytin during whole-cell patch clamp recording. The insets show the coexpression of Cre-GFP (green) and PV (red) in both neurons (arrows). *Gcl* granule cell layer, *ml* molecular layer, *Bio* biocytin. (**f**) Quantification of dendritic length based on reconstructions of the dendritic arbour. (**g**) Sholl analysis applied to the dendrites of fast-spiking interneurons of control and HPC-Lhx6^−/−^ mice. (**h**) Top, confocal image stack of DG granule cells filled with biocytin during whole-cell recording. PV is shown in red, Cre-GFP in green. Bottom, examples and summary of minimal stimulation in the gcl to evoke perisomatic inhibitory postsynaptic currents (IPSCs). There was no difference in IPSC amplitude. (**i**) Facilitation indices obtained from the amplitudes of IPSCs evoked at different interstimulus intervals were unaltered in HPC-Lhx6^−/−^ granule cells. (**j**) Example of unaltered multiple-pulse behavior in response to trains of 10 pulses at 50 Hz. Data points show averages for individual cells. Data are mean ± sem. Schematic in (**h**) was created with Inkscape 0.92 (www.inkscape.com).

Adult PVIs are characterized by a fast-spiking action potential phenotype that undergoes maturation during postnatal development^15^. In contrast, Lhx6-deficient MGE cells transplanted into wildtype host mice show a late-spiking phenotype^9^, suggesting that Lhx6 might be required to develop fast-spiking properties. To test whether Lhx6 is required to maintain a mature physiological phenotype of PVIs in adult mice, we performed whole-cell patch clamp recordings from PVIs in acute slice preparations of the DG of 13 weeks-old mice. PVIs of both control and HPC-Lhx6^−/−^ mice discharged at high frequency in response to somatic current injection (Fig. 3a). There was no difference in the action potential rate at any tested current amplitude (150–700 pA) or maximal firing frequency between both groups (106.0 ± 12.1 vs. 112.9 ± 7.9 Hz maximal firing rate for control and HPC-Lhx6^−/−^ mice, respectively, p = 0.634, t-test, N = 7 and 8 cells, Fig. 3b). Consistent with unaltered high-frequency firing, the input resistance did not differ between PVIs from both groups (193.4 ± 29.3 vs. 157.0 ± 11.0 MΩ, p = 0.336, Mann–Whitney U-test, N = 7 and 8 cells, Fig. 3d). Moreover, the properties of individual action potentials were unchanged between the two groups (amplitude 86.4 ± 1.8 vs. 87.1 ± 1.9 mV, p = 0.56; half-width 0.55 ± 0.05 vs. 0.53 ± 0.03 ms, p = 1; rise time 0.16 ± 0.01 vs. 0.17 ± 0.004 ms; for control and HPC-Lhx6^−/−^ mice respectively, p = 0.24; Mann–Whitney U-tests, N = 7 and 8 cells, Fig. 3c,d). These results argue against changes in active or passive membrane properties upon removal of Lhx6 in adulthood.

We further assessed potential alterations in morphology by quantifying dendritic length of biocytin-filled fast-spiking interneurons (Fig. 3e,f). Total dendritic length did not differ between PVIs from control and HPC-Lhx6^−/−^ mice (3.2 ± 0.1 vs. 3.3 ± 0.3 mm, p = 0.874, N = 6 and 8 neurons, Fig. 3f). Furthermore, Sholl analysis revealed no difference in the number of dendritic grid crossings over a distance up to 200 µm from the soma (Fig. 3g).

Finally, we analyzed whether the synaptic output of PVIs was altered after removal of the Lhx6 gene. We stimulated PVI axons by placing an extracellular stimulation electrode at the granule cell layer of the DG, and observed the evoked inhibitory response (excitation was blocked with 4 mM Kynurenic acid) in granule cells. When stimulation intensity was set to evoke the smallest visible IPSCs, we observed a similar amplitude in control and HPC-Lhx6^−/−^ animals (− 43.8 ± 6.9 vs. − 44.8 ± 4.8 pA, p = 0.9, unpaired t-test, N = 12 and 11 granule cells, Fig. 3h). Moreover, synaptic properties of PVIs were also unaltered by our manipulation as both mice showed similar paired-pulse facilitation (i.e., facilitation index with 10 ms interstimulus interval was 0.98 ± 0.05 vs. 1.06 ± 0.07, p = 0.55, N = 12 and 11 granule cells, two-way ANOVA, Fig. 3i) and synaptic depression in a 10 pulses (50 Hz) stimulation protocol (facilitation index at the 10th pulse was 0.34 ± 0.05 vs. 0.27 ± 0.03, p = 0.45, unpaired t-test, N = 12 and 11 granule cells for control and HPC-Lhx6^−/−^, respectively, Fig. 3j). Jointly, these data suggest that adult knockdown of Lhx6 does not influence key physiological or morphological features of PVIs.

### Unaltered cognitive performance upon removal of Lhx6 from adult PVIs

**Figure 4:**
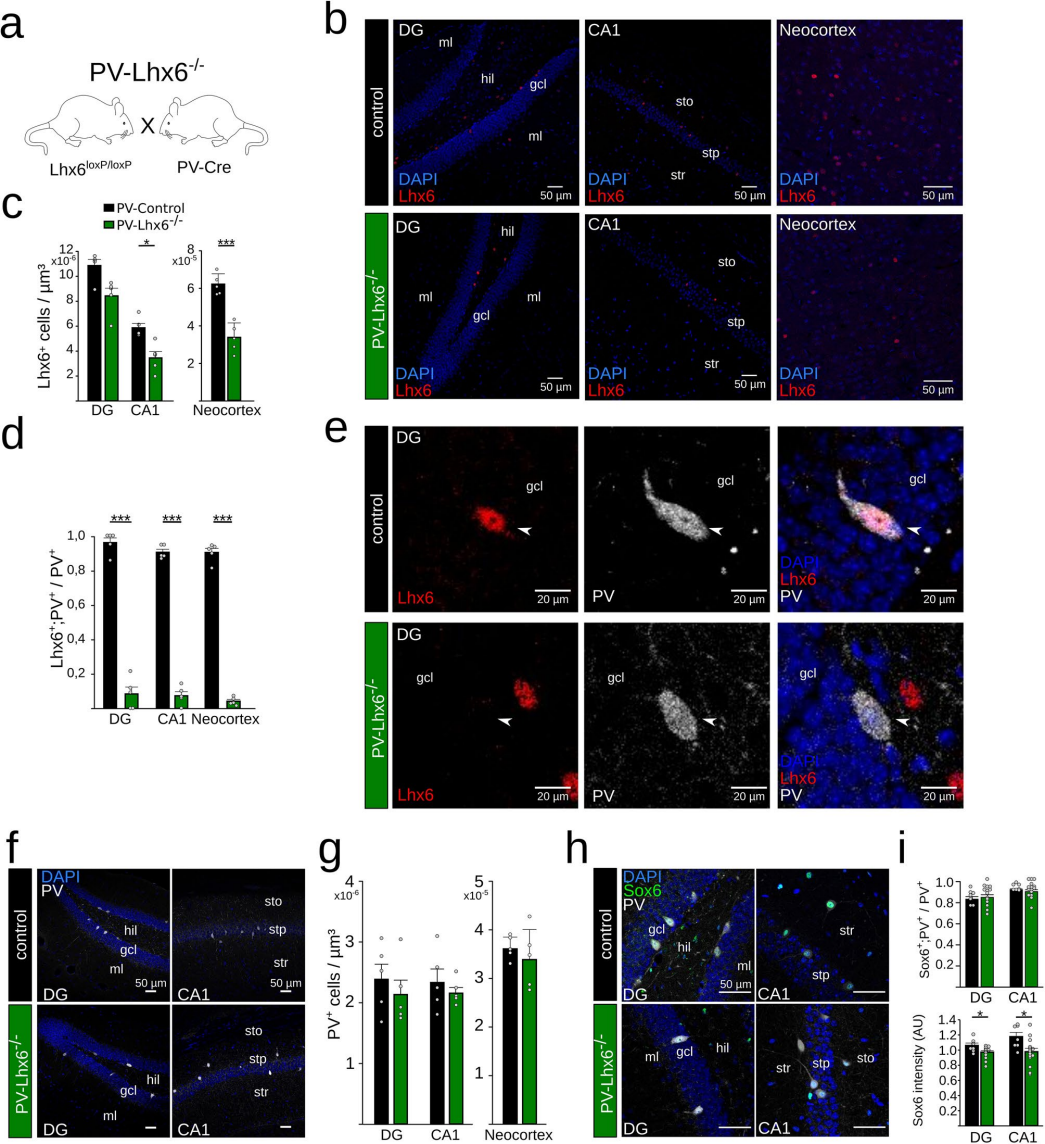
Removing Lhx6 during postnatal development. (**a**) Schematic of the experimental procedures used to remove Lhx6 from cortical PVIs: Mice expressing Cre-recombinase under the control of the PV-promotor were crossed with Lhx6^loxP/loxP^ mice. These animals are referred to as PV-Lhx6^−/−^ mice. (**b**) Confocal image stacks showing the expression of Lhx6 (red) and DAPI (blue) in DG (left), CA1 (middle) and neocortex (right). (**c**) Quantification of the number of Lhx6-positive cells. (**d**,**e**) Quantification and confocal examples of the colocalisation of Lhx6 and PV in control and PV-Lhx6^−/−^ mice. The proportion of PVIs expressing Lhx6 was significantly reduced in DG, CA1 and neocortex. (**f**) Overview images used to quantify the expression of PV in PV-Lhx6^−/−^ and control mice. (**g**) Unaltered number of PVIs in DG, CA1 and neocortex. (**h**) Costaining of Sox6 (green), PV (white) and DAPI (blue). (**i**) Unaltered number of Sox6-positive neurons (top) and mild reduction in Sox6 intensity (bottom). Data points show mouse averages. Data are mean ± sem. *p < 0.05, ***p < 0.001. Schematic in (**a**) was created with Inkscape 0.92 (www.inkscape.com).

Next, we asked whether removal of Lhx6 from adult PVIs might impact cognitive functions. To achieve maximal sensitivity, we generated a brain-wide Lhx6-knockdown model by crossing mice expressing Cre recombinase under the control of the PV promoter with Lhx6^loxP/loxP^ animals (we refer to these mice as PV-Lhx6^−/−^, Fig. 4a). The PV promoter is only activated during the second postnatal week, sparing PVIs during prenatal and early postnatal development from Cre expression and Lhx6 removal^16–18^. Importantly, the recombination and thus the knockdown of Lhx6 is not restricted to the hippocampus^19,20^. Postnatal Cre-dependent knockout in PV-Lhx6^−/−^ animals produced similar effects compared to the AAV-dependent approach. First, we found a significant reduction in the total number of Lhx6^+^ cells in CA1 and a trend in the DG compared to 12 week old PV-Cre control animals (DG: 10.8 ± 0.4 × 10^–6^ versus 8.38 ± 0.6 × 10^–6^ Lhx6^+^ cells/µm^3^, p = 0.056; CA1: 5.8 ± 0.3 × 10^–6^ versus 3.43 ± 0.5 × 10^–6^ Lhx6^+^ cells/µm^3^, p = 0.004, unpaired t-tests, n = 1 slice per animal, N = 5 mice each, Fig. 4b,c). In addition, a significant reduction in the number of Lhx6-positive cells was observed in the neocortex (6.2 ± 0.2 × 10^–5^ and versus 3.3 ± 0.3 × 10^–5^ cells/µm3, p < 0.001, unpaired t-test, n = 2 slices per animal, N = 5 mice each, Fig. 4b,c). Second, we found a strong reduction in the proportion of hippocampal PVIs expressing Lhx6 (DG: 96.1 ± 2.8 versus 8.5 ± 4.0%; CA1: 90.6 ± 1.7 versus 7.4 ± 2.4% for control and PV-Lhx6^−/−^ mice respectively, p < 0.001, unpaired t-tests, n = 1–2 slices per animal, N = 5 mice each) and in the neocortex (4.2 ± 1.0% vs. 90.5 ± 2.4%, p < 0.001, unpaired t-test, n = 1–2 slices per animal, N = 5 mice each, Fig. 4d,e), indicating efficient widespread removal of the Lhx6 protein from cortical PVIs. In contrast, Lhx6 expression was readily observable in PV-negative cells supporting the specificity of the knock-down (Fig. 4e). Third, similar to the HPC-Lhx6 knockout, PV-Lhx6^−/−^ mice had the same number of PVIs as control animals (DG: 2.3 ± 0.6 × 10^–6^ versus 2.1 ± 0.5 × 10^–6^ PV^+^ cells/µm^3^, p = 0.334; CA1: 2.3 ± 0.2 × 10^–6^ versus 2.2 ± 0.2 × 10^–6^ PV^+^ cells/µm^3^, p = 0.4, unpaired t-tests; Neocortex: 3.6 ± 0.1 × 10^–5^ vs. 3.4 ± 0.3 × 10^–5^, p = 0.548, Mann–Whitney-U-Test, n = 1 slice per animal, N = 5 mice each, Fig. 4f,g), demonstrating unaltered survival of PVIs. Finally, the proportion of PVIs expressing Sox6 was unchanged after Lhx6 knockdown in these mice (DG: 83.2% vs. 85%, p = 0.682; CA1: 92.3% vs. 90.5% p = 0.533, unpaired t-tests, n = 1 slice per animal, N = 7 and 15 mice Fig. 4h,i) while a mild decrease in Sox6 immunolabeling was observed (DG: 1.06 ± 0.03 vs. 0.97 ± 0.02, p = 0.03; CA1: 1.18 ± 0.06 vs. 0.98 ± 0.04, p = 0.017, unpaired t-tests, n = 1 slice per animal, N = 7 and 15 mice, Fig. 4i), similar to what we observed in HPC-Lhx6^−/−^ mice. Therefore, using PV-Lhx6^−/−^ mice, Lhx6 is reliably removed from hippocampal and neocortical PVIs during postnatal development.

**Figure 5:**
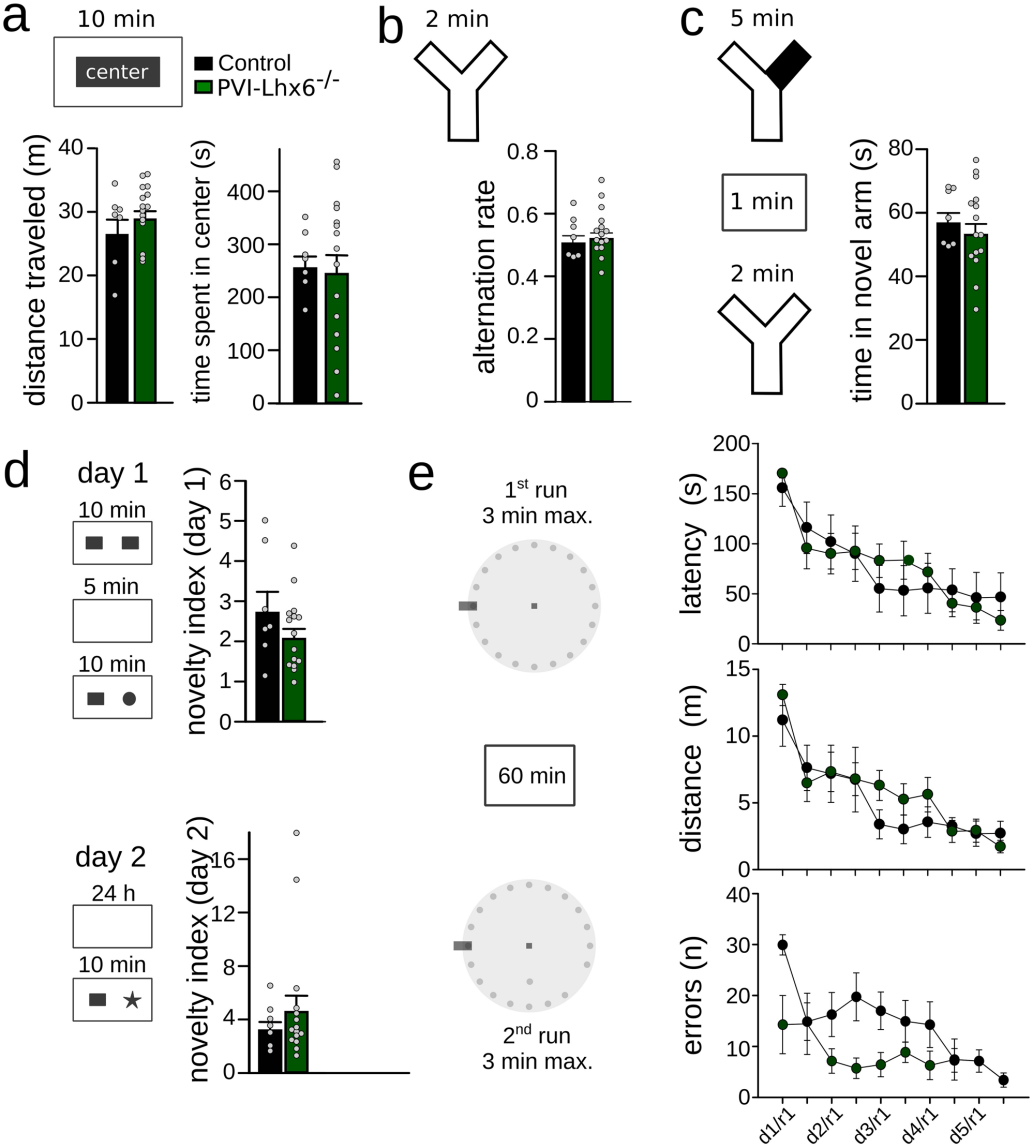
Intact cognitive behavior upon postnatal removal of Lhx6. (**a**) Open field task. PV-Lhx6^−/−^ mice displayed similar total distance travelled (left) and time spent in the center of the arena (right), indicating unaltered motor behavior and anxiety levels. (**b**) Spontaneous alternation task. There was no significant difference in the alternation rate of PV-Lhx6^−/−^ mice. (**c**) Novel arm exploration task. Left: schematic of the procedure. Right: during the test phase, PV-Lhx6^−/−^ mice spent equal times in the novel as control animals. (**d**) Novel object task. Left. Schematic of the procedure. Right: quantification of the novelty index for 5 min (top) and 24 h delay periods (bottom) indicated unchanged object recognition in PV-Lhx6^−/−^ mice. (**e**) Barnes maze task. Left: schematic of the test. Right: quantification of latency to the escape tunnel (top), distance travelled (middle) and errors (bottom) revealed intact spatial learning in PV-Lhx6^−/−^ mice. Data points show mouse averages. Data are mean ± sem. Schematics were created with Inkscape 0.92 (www.inkscape.com).

First, to control for possible effects of Lhx6 knock-down on anxiety and locomotion, we exposed the animals to an open field arena and quantified the time spent in the center of the maze. There was no detectable difference between groups, suggesting unaltered basal anxiety levels (Fig. 5a). Furthermore, the total distance travelled did not differ between groups, indicating unaltered motor behavior (Fig. 5a).

Because PVIs are necessary for working memory^21,22^, we tested whether removal of Lhx6 might affect the ability to execute working memory. Abolishing the expression of Lhx6 in PVIs from adult animals had no influence on the performance in a spontaneous alternation task (proportion of alternations: 0.50 ± 0.03 vs. 0.52 ± 0.02, p = 0.677, unpaired t-test, N = 7 (2 males und 5 females) and 15 mice (9 males and 6 females), in control and PV-Lhx6^−/−^ respectively, Fig. 5b). Furthermore, we tested short-term memory in a novel arm task, in which the animals initially explore two accessible arms of a Y-maze and are re-exposed to the maze with all three arms open after a delay of 1 min. During re-exposure, mice typically spend more time in the previously inaccessible arm during the recall phase^23^. We detected no significant difference between groups (56.5 ± 3.3 and 52.8 ± 3.5 s spent in the newly accessible arm for control and PV-Lhx6^−/−^ mice, respectively, p = 0.527, unpaired t-test, N = 7 and 15 mice, Fig. 5c), pointing to intact short-term memory in PV-Lhx6^−/−^ mice.

Experimentally reducing excitatory drive to PVIs impairs object recognition^24^. We therefore tested whether the lack of Lhx6-expression in PVIs might affect this type of memory. In a novel object recognition task, control and PV-Lhx6^−/−^ mice showed the same level of enhanced interest for a novel object for short (5 min 2.70 ± 0.53 and 2.05 ± 0.24, p = 0.21, t-test, N = 7 and 15 mice, values are preference indices) or long delays between encoding and retrieval (24 h, 3.15 ± 0.63 and 4.53 ± 1.23, p = 1.0, Mann–Whitney U-test N = 7 and 15 mice Fig. 5d).

PVIs have furthermore been reported to be fundamental for spatial learning^25,26^. We therefore evaluated whether adult mice lacking Lhx6 show deficits in the Barnes maze task, in which animals learn the location of a hidden escape hole over successive days (Fig. 5e). Both groups of mice learned the location of the escape tunnel during the 5-day protocol without significant differences between groups (time: p = 0.934, t = 0.0843; distance: p = 0.475, t = 0.728; errors p = 0.247, t = 1.194, N = 7 control and 15 PV-Lhx6^−/−^ mice, Two-Way Repeated Measures ANOVA, Fig. 5e). In summary, these data indicate that postnatal Lhx6 in PVIs is not required for a wide range of cognitive behaviors in adult mice.

## Discussion

In this study, using two conditional knockout models to probe the functional consequences of postnatal Lhx6 loss, we found preserved MGE-derived interneuron numbers in the hippocampus and neocortex, unchanged functional properties of PVIs and unaltered cognitive behavior upon removal of Lhx6 from PVIs during postnatal stages. As a result of adult Lhx6-knockdown, the expression of downstream targets that depend on Lhx6 during early development is reduced.

Prenatal Lhx6 knockdown is associated with the loss of expression of downstream transcription factors, including the proteins investigated in this study^7,14,27^. Arx expression is strongly reduced as soon as MGE interneurons lacking Lhx6 start migrating towards the cortex^7,28^. Similarly, Sox6 expression is undetectable in more than 80% of the interneurons in Lhx6 knockout mice^7,14,27,28^. These changes in gene expression lead to a strong reduction in the number of MGE-derived interneurons in the cortex and hippocampus due to insufficient differentiation and migration of interneuron precursors^6,7^. As a result, mice develop general weakness and epilepsy shortly after birth and die within ~ three weeks^6,7^. In contrast, we observed that loss of Lhx6 at mature stages, when migration and differentiation of interneurons are already completed, has no apparent effect on the amount and functional properties of mature interneurons. This observation is consistent with previous reports showing only mild or no effects at all after postnatal removal of other transcription factors which are fundamental for proper prenatal development of cortical interneurons. Knockdown of the transcription factors Dlx1&2 around P10 does not affect the number of PVIs in the cortex while embryonic knockout leads to a reduction in PVI count by ~ 50%^29^. Similarly, embryonic knockout of Satb1 leads to reduced numbers of SOMIs in the cortex whereas the SOMI distribution was not affected even when the knockout was performed on the first postnatal day^30^. Finally, removal of Sox6 from cortical SOM interneurons decreases cell type heterogeneity without changing total cell numbers or the expression of downstream targets^31^. These findings, together with our results, suggest that transcription factors controlling early interneuron development might become irrelevant for the survival and function of interneurons at postnatal stages.

The Cre-lox system used in this work has been frequently used as a tool for creating conditional and viable knockout models of genes involved in interneuron specification^29–31^, where successful Cre-dependent removal of interneuron-specific genes was repeatedly demonstrated using in-situ hybridization, single cell RNA-sequencing or immunohistochemistry. We observed successful removal of Lhx6 in > 90% of adult PVIs, while a small percentage of neurons still expressed Lhx6 with unchanged immunolabelling intensity. This suggests that recombination, although highly efficient, did not occur in every Cre-expressing interneuron. Because the PV-Cre transgenic line does not express the recombinase in the entire population of target interneurons, remaining Cre-expression in a subset of PVIs in PV-Cre mice could explain the slightly higher efficiency of our viral knockdown approach (Figs. 1 and 4). Nevertheless, expression analysis of the downstream target Sox6 was performed in PVIs in which Lhx6 removal was confirmed, suggesting that continued expression of Lhx6-downstream targets is not due to a lack of efficiency in recombination but to postnatal changes in the interaction mechanisms between these transcription factors. Moreover, we observed significant, although moderate changes in the expression level of these proteins, further confirming that our intervention to postnatally remove Lhx6 was successful. Notably, the effects of Lhx6 removal on downstream targets was similar in viral-induced adult and in postnatal knock-down of Lhx6 in PV-Cre transgenic mice. Since the expression of Cre-recombinase starts during the second postnatal week^16–18^, these results suggest that Lhx6 becomes dispensable for PVI maturation already during the second to third postnatal week. However, we have focused on behavioral and transcriptional effects in adult Lhx6-deficient mice, and further studies are required to reveal the exact time course of the uncoupling of Lhx6 from its downstream partners.

It is remarkable that knockdown of Lhx6, a gene which during early developmental stages is necessary for survival^6^, has no effect in any of the behavioral tests performed. More so, when using the transgenic PV-Cre line^19^ to drive knock-down of Lhx6, recombination is expected in different brain regions^20^ involved in locomotion, anxiety and learning. Nevertheless, when we consider that the expression of Lhx6-downstream targets was only slightly reduced and that DG PVIs conserved their main morphological and physiological properties, these results suggest that adult PVIs remain capable of fulfilling their functional roles in the absence of Lhx6, resulting in overall intact circuit function and behavior.

Why is Lhx6 essential for interneuron development during early stages but seemingly dispensable later in life? It is possible that transcriptional networks deciding interneuron fate undergo postnatal changes that result in the emergence of degenerate and redundant transcriptional properties. In the framework of degeneracy, removing a key element from a complex connected system might be compensated by interactions within the network^32,33^. For example, the loss of ‘essential’ gene products does not always lead to fatal consequences for the organism, as underscored by viable mice lacking myoglobin^34^ or healthy human individuals without detectable serum levels of albumin^35^. Furthermore, in the central nervous system, some transcription regulatory elements have a redundant function^36–38^. Similarly, although Lhx6 is a crucial part of a causal chain of transcription factors driving interneuron migration and specification^6,7,10^, other transcription factors might be sufficient to drive the expression of downstream genes in the adult brain. This implies that the transcriptional network undergoes developmental changes resulting in stabilization and increased degenerate properties. Developmental changes in transcriptional networks have been shown for example in the case of Sox2, which changes its downstream target genes during the differentiation from embryonic to trophoblastic stem cells^39,40^ or in the case of Pomc enhancers NEP1 and NEP2 whose function changes from being synergistic to additive, making NEP2 function redundant^41^. Further studies are required to understand network stabilization and the potential emergence of degenerate properties during the postnatal phase.

What would be the function of a redundant transcription factor network in adult interneurons? Epigenetic reduction of Lhx6 is related to cell proliferation and tumor formation in glia cells, hepatocytes and epithelial cells of the lung^42–44^, suggesting that Lhx6 is required to maintain cellular differentiation in non-neuronal tissues. Neurons are terminally differentiated cells that remain largely stable during adulthood^45^. A redundant set of transcription factors in adulthood might thus function as a failsafe mechanism to maintain interneuron differentiation and survival. Since GABAergic cortical interneurons fulfill several critical roles including the generation of brain rhythms and maintenance of proper excitation/inhibition balance in healthy subjects^46–48^, the robust differentiation state of interneurons might be a crucial prerequisite for cortical circuit function.

## Methods

### Animals

8–20 week old mice of both sexes with free access to food and water were used in this study. Lhx6^loxP/loxP^ mice were kindly provided by Vassilis Pachnis (The Francis Crick Institute, London, UK). C57Bl6/J and PV-Cre mice were used as controls. To generate PV-Lhx6^−/−^ mice, Lhx6^loxP/loxP^ mice were crossed with mice expressing Cre under the control of the PV promoter (PV-Cre mice, Jax line 017320). Experiments were carried out in accordance with national legislation and were approved by the animal ethics committee of the Regional Administrative Council (Regierungspräsidium) Freiburg.

### Surgical procedures

8 week old mice were deeply anesthetized with isoflurane (induction: 3.5% in oxygen, maintenance: 1.5–2.5%). Buprenorphine (0.05–0.1 mg/kg body weight) was injected subcutaneously for pain relief and the animals were placed on a heating pad in a stereotaxic apparatus (model 1900, Kopf Instruments). A small craniotomy was performed above the dorsal hippocampus at 1.75 mm posterior and 2.0 mm lateral of bregma. Viral injections into the dorsal hippocampus were performed at two depths (1.5 mm and 2.3 mm) to maximize infection. For in vitro electrophysiology, injections were targeted to the ventral hippocampus at 2.55 posterior and 2.9 lateral of bregma. Injections were performed at 4 levels in this case (2.9, 2.6, 2.3 and 2.0 below bregma). A volume of 0.5–0.8 µl of Cre-GFP-encoding virus (AAV9-CMV-eGFP-Cre, Penn Vector Core, original titer 9.82 × 10^12^/ml, diluted 1:500 in phosphate buffered saline (PBS)) was injected per depth at a rate of approximately 0.1 µl/min using a Hamilton syringe. After injection, the syringe was kept in place for 5 min to allow viral diffusion into the tissue and then retracted. The craniotomy was sealed with bone wax (Bone Wax, Surgical Specialties Corporation) and the skin closed with tissue glue (Vetbond, 3 M). For postoperative analgesia, buprenorphine was injected subcutaneously for 3 days (3 injections/day) and supplied in the drinking water overnight.

### Immunohistochemistry

Mice were deeply anesthetized with an intraperitoneal injection of urethane (2 mg/kg body weight) and intracardially perfused with PBS followed by 4% paraformaldehyde (PFA; 10–13 min). After postfixation in PFA overnight, frontal 60 µm-thick sections of the hippocampus were cut with a vibratome (VT 1000-S, Leica). The slices were washed in PBS, permeabilized in 0.4% TritonX-100 (Sigma-Aldrich) for 30 min, blocked in 0.2% TritonX-100 and 4% normal goat serum (NGS, Jackson ImmunoResearch) for 30 min, and incubated with primary antibody solution composed of 0.1% TritonX-100 and 2% NGS overnight at 4 °C. Primary antibodies used in this study were rabbit-anti-Lhx6 (gift from Vassilis Pachnis, London, 1:1000), rabbit-anti-PV (Swant, 1:1000), guinea-pig-anti-PV (Swant, 1:1000), mouse-anti-PV (Swant, 1:1000), guinea pig-anti-Sox6 (gift from Michael Wegner, Erlangen, 1:1000) and rabbit-anti-Arx (gift from Ken-ichirou Morohashi, Kyushu, 1:500). After three washes in PBS containing 1% normal goat serum, secondary antibodies were applied in PBS and 1.5% NGS for 2.5–3 h. Secondary antibodies used were Cy3-anti-rabbit (Jackson ImmunoResearch, 1:1000), AlexaFluor647-anti-guinea pig (Invitrogen, 1:1000) and AlexaFluorPlus555-anti-mouse (Invitrogen, 1:1000). After 2 washing steps, the slices were incubated in 0.1% 4ʹ,6-diamidine-2-phenylindol for 5 min. After 3 more washing steps, the slices were mounted in Mowiol (Mowiol 4–88, Roth).

For immunostaining of acute slices after in vitro electrophysiology (see below), the sections were fixed in 4% PFA overnight, washed 3 times in PBS (15 min), and blocked in 10% NGS for 60 min. Primary antibody (rabbit-anti-PV, 1:1000) was incubated in PBS containing 0.3% TritonX-100 and 5% NGS for 24 h at room temperature. Secondary antibody (Cy3-anti-rabbit, 1:1000) was applied along with StreptavidinAlexaFluor647 (1:500) for 24 h at room temperature. After 3 washing steps in PBS, DAPI was applied for 5 min as above. After 3 additional washes the sections were embedded in Mowiol.

### Image acquisition and analysis

Confocal image stacks were acquired with a laser scanning microscope (LSM 710 or 900, Zeiss). For expression and colocalisation analyses, a 20 × objective (Plan-APOCHROMAT, NA 0.8) was used. A 63 × objective was used for high magnification images (Plan-APOCHROMAT, NA 0.7). Analysis was performed on slices with confirmed expression in the DG/CA1.

Image analysis was performed using ImageJ routines. To measure cell densities, the number of neurons in the hippocampal area under investigation was determined visually using the CellCounter plugin. The neuron counts were divided by the 3-d volume estimated from confocal stacks. Fluorescence intensities were determined in a single z-level containing the nucleus of the neuron as identified by DAPI-staining. For each neuron, background staining was determined in the neuropil surrounding the cell. Fluorescence intensity of each neuron was normalized to the background measurement for that cell. To account for differences in labelling intensity, the background-subtracted values were further normalized to the expression intensity in PV-negative neurons of the same section, respectively. This procedure allowed the objective measurement of labelling intensity. No additional attempts to randomize the mice used for the experiments were made. Quantification of Lhx6-expression in neocortical PVIs was performed in the posterior parietal cortex.

### In vitro electrophysiology

Patch-clamp recordings in hippocampal slices were carried out in HPC-Lhx6^−/−^ and control mice 5 weeks after viral injection (age: 13 weeks) as described before^13^. In brief, the animals were anesthetized in isoflurane (3.5% in oxygen) and decapitated. Transverse 300 µm-thick slices of the ventral hippocampus were cut with a vibratome (VT1200, Leica) in a chilled solution containing (in mM): 87 NaCl, 25 NaHCO_3_, 2.5 KCL, 1.25 NaH_2_PO4, 10 glucose, 0.5 CaCl_2_, 7 MgCl_2_ and 75 sucrose aerated with 95% O_2_, 5% CO_2_. The sections were then transferred to oxygenated artificial cerebrospinal fluid (ACSF) containing (in mM): 125 mM NaCl, 25 mM NaHCO_3_, 2.5 mM KCL, 1.25 mM NaH_2_PO4, 25 mM glucose, 2 mM CaCl_2_, 1 mM MgCl_2_, recovered for 30 min at 34 °C and then stored at room temperature until use.

Whole cell patch-clamp recordings were performed with an Axopatch 200B or Multiclamp 700B amplifier using custom software (F-pulse, running under Igor Pro 5). Recordings were targeted to Cre-GFP-expressing neurons in the DG using 488 nm light illumination (CoolLED pE-100, Ludesco). Cre-GFP-positive neurons with characteristic morphological features resembling PVIs (large soma size compared to DG granule cells) were patched for recording using pulled glass pipettes (resistance 2–5 MΩ). An internal solution composed of (in mM) 120 K-Gluconate, 20 KCl, 10 EGTA, 2 MgCl_2_, 2 Na_2_ATP, 10 HEPES, 0.2% biocytin (pH 7.26 adjusted with KOH, ~ 300 mOsm) was used.

To determine action potential firing properties, current injections of increasing amplitude (− 100 to 1250 pA, 50 pA increments, 1 s duration) were made in current clamp while a constant current was injected to hold the cell at − 70 mV. Input resistance was determined using Ohm’s law from the steady-state response to a 1 s-long 10 mV pulse applied in voltage clamp from a holding potential of − 70 mV. Patch-clamp data were analysed using Stimfit^49^. Biocytin was included in the pipette to allow post-hoc visualisation of the cellular morphology. Recordings with an access resistance of up to 23 MΩ were accepted for analysis. All recordings were conducted at room temperature. No randomization/blinding was applied to the in vitro experimental groups.

### Behavioral experiments

Behavioral tests were carried out with PV-Lhx6^−/−^ mice (9 males and 6 females) and PV-Cre mice (2 males and 5 females) with the experimenter blind to the genotype of the animals. The movement of the animals was recorded with a camera and tracked using EthoVision (Noldus). Behavioral apparatuses were cleaned with 70% ethanol between subjects and trials.

To assess behavior in the open field, the animals were placed in a 40 × 25 cm box for 10 min. Spontaneous alternation was measured in a Y-maze (30 cm arm length, 8 cm width, 15 cm height, 60° angles between arms). Exploration time was 5 min. An arm entry was counted if both front paws entered the arm. Alternation rate was calculated as the number of observed alternations divided by the possible number of alternations. Novelty preference was carried out in the same Y-maze as spontaneous alternation with one of the arms initially blocked off. The mice were allowed two explore the arena for 5 min and were then returned for 1 min to their holding cage. Afterwards, the mice were re-exposed to the Y-maze for 2 min with all three arms accessible and the time spent in the novel arm during re-exposure was quantified. Novel object recognition was performed one day after the open field test in the same arena. Two large red c-shaped magnets were placed in the arena and the animals were allowed to explore the objects for 10 min. Afterwards, they were returned to their holding cage for 5 min and then re-exposed to the arena for 10 min. During re-exposure, one of the objects was replaced by a green w-shaped clamp. 24 h later, another re-exposure session (10 min) was performed, during which a black triangle-shaped object was used. Novelty index was calculated as the time spent with the exchanged relative to the familiar object. Only time periods when the mouse was directly interacting with the object (orientation towards the object or touching the object with the front paws) were counted. Sitting on the object did not count as an interaction. For Barnes maze learning, the animals were placed in the center of a brightly lid circular arena (100 cm diameter) with 20 holes (5 cm) around the edge, only one of which was equipped with an escape box underneath. The animal was allowed to search for the escape box for a maximum of 3 min, after which it was guided to the escape hole by the experimenter. After finding the escape box, the animal was kept in the box for ~ 30 s and then returned to the holding cage. 2 runs were performed per day for 5 consecutive days, with 60–70 min between individual runs. Latency to reach the target, as well as distance traveled, were quantified.

### Statistical analysis

Statistical tests were performed using Sigma Plot 12 (Systat). For the comparison of two groups, an unpaired two-sided *t*-test was used in case of data following a normal distribution, otherwise a Mann–Whitney U-test was used. Shapiro–Wilk test was used to assess normality of the data. Multiple comparisons were performed with a one-way ANOVA followed by Holm-Sidak post-hoc testing. To compare two groups over several measurement time points we used a two-way repeated measures ANOVA. Significance level was set to 95%. The data in this study are reported in accordance with ARRIVE guidelines.

## Supplementary Information

Supplementary Information.
