# Supplementary material for: Interneuron function and cognitive behavior are preserved upon postnatal removal of Lhx6
Source: Sci Rep. 2022 Mar 22;12:4923. doi: 10.1038/s41598-022-09003-4 (PMC8941127; doi:10.1038/s41598-022-09003-4)
Supplement: Supplementary file 1 — Supplementary Information. [file 41598_2022_9003_MOESM1_ESM.pdf]

Supplementary Information

Interneuron function and cognitive behavior are preserved upon postnatal removal of Lhx6.

Supplementary Figures 1-2

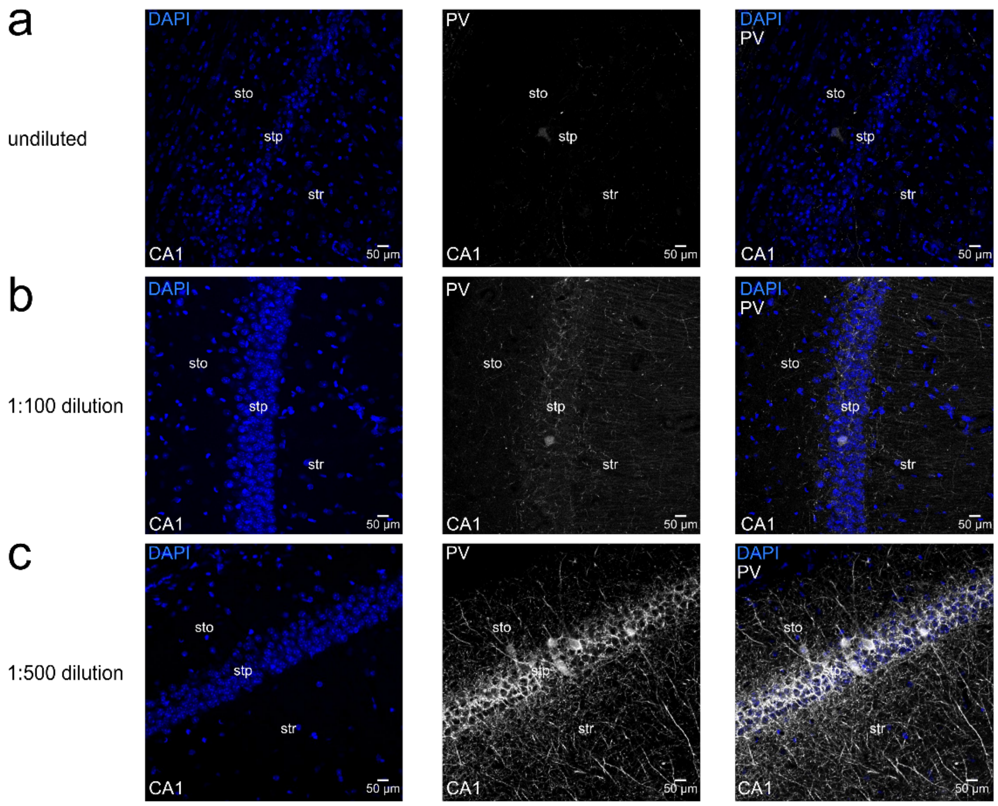

Supplementary Figure 1: Effects of injections with different concentrations of AAV9-CRE-GFP. **(a)** Confocal image stacks of DAPI (blue, left), PV-staining (white, middle) in CA1 of C57/BL6-mice 5 weeks after injection of undiluted AAV9-CRE-GFP (corresponding to a titer of $9.8*10^{12}$/ml). sto: stratum oriens; stp: stratum pyramidale; str: stratum radiatum. In the DAPI staining a narrowing of the stratum pyramidale and a hypercellularity in the stratum oriens and stratum radiatum are visible. The PV signal intensity and the number of PV+ interneurons are strongly reduced. **(b)** same as (a) but after injection of AAV9-CRE-GFP diluted 1:100 with PBS. A narrowing of the stratum pyramidale is not visible. Hypercellularity and reduction of the PV signal are visible but to a lesser extent. **(c)** same as (a and b) but after injection of AAV9-CRE-GFP diluted 1:500 with PBS. Using this dilution preserves circuit anatomy and immunoreactivity.

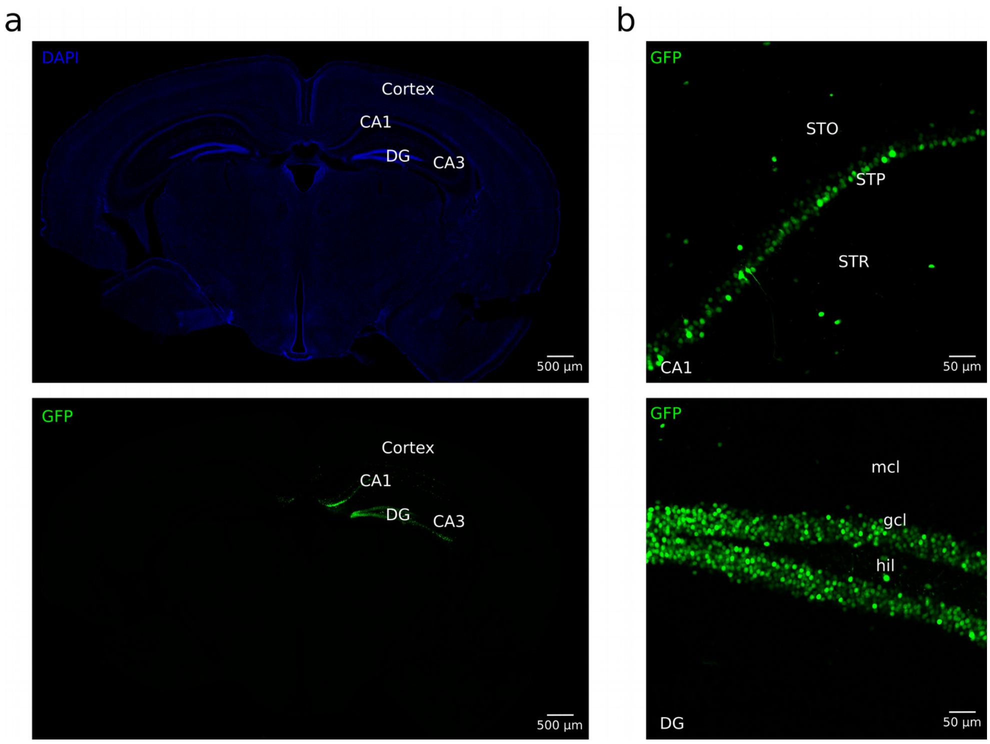

Supplementary Figure 2: Expression of Cre-GFP in the hippocampus. (a) Example confocal image stacks showing the expression of Cre-GFP (green) and DAPI (blue). Note that expression is mostly restricted to DG and CA1. **(b)** Higher magnification image stack of CA1 (top) and the DG (bottom).
